# Supplementary material for: Effects of food-based enrichment on enclosure use and behavioral patterns in captive mammalian predators: a case study from an Austrian wildlife park
Source: PeerJ. 2023 Oct 9;11:e16091. doi: 10.7717/peerj.16091 (PMC10569183; doi:10.7717/peerj.16091)
Supplement: Supplemental Information 1 [file peerj-11-16091-s001.docx]

**PeerJ**

**Supplementary Material**

Effects of food-based enrichment on enclosure use and behavioral patterns in captive mammalian predators: a case study from an Austrian wildlife park

Verena Puehringer-Sturmayr^1,2^, Monika Fiby^3^, Stephanie Bachmann^1^, Stefanie Filz^1^, Isabella Grassmann^1^, Theresa Hoi^1^, Claudia Janiczek^1^, *Didone Frigerio^1,2^

Authors’ affiliations and current addresses:

^1^Core Facility Konrad Lorenz Research Center for Behavior and Cognition, University of Vienna, Grünau im Almtal, Austria

^2^Department of Behavioral and Cognitive Biology, University of Vienna, Vienna, Austria

^3^Zoo Design and Consulting, Vienna, Austria

*Corresponding author: Didone Frigerio

Fischerau 13, Grünau im Almtal, 4645, Austria

Email address: [didone.frigerio@univie.ac.at](mailto:didone.frigerio@univie.ac.at)

Figure S1. Proportion of visibility in brown bears, separately for each combination of phase (pre-enrichment, during enrichment) and time of day interval (yellow: 0800 AM – 1100 AM, grey: 1100 AM – 0200 PM, blue: 0200 PM – 0600 PM). Bubbles indicate the number of observations in the respective combination.
